# Association between triglyceride-glucose related indices and at-risk NASH in U.S. adults with NAFLD: results from NHANES 2017-2020

**DOI:** 10.3389/fendo.2025.1604991

**Published:** 2025-06-04

**Authors:** Hanchen Ma, Wenjie Zhang, Zheng Zhao, Shuaichen Jin, Linchuan Li, Ruizhao Zong, Wuyang Li, Ziwei Zhang, Shichang Cui, Yunmiao Pan, Jiankang Zhu, Guangyong Zhang

**Affiliations:** ^1^ Department of General Surgery, Shandong Provincial Qianfoshan Hospital, Cheeloo College of Medicine, Shandong University, Jinan, China; ^2^ Department of General Surgery, The First Affiliated Hospital of Shandong First Medical University & Shandong Provincial Qianfoshan Hospital, Jinan, China; ^3^ Laboratory of Metabolism and Gastrointestinal Tumor, The First Affiliated Hospital of Shandong First Medical University & Shandong Provincial Qianfoshan Hospital, Jinan, China

**Keywords:** nonalcoholic fatty liver disease, nonalcoholic steatohepatitis, insulin resistance, triglyceride-glucose index, body fat distribution, NHANES

## Abstract

**Introduction:**

Nonalcoholic fatty liver disease (NAFLD), intricately linked to insulin resistance (IR), obesity, and metabolic syndrome (MetS), affects nearly one-third of the global population. The triglyceride-glucose (TyG) index and its derivative metrics, which integrate anthropometric measures, have emerged as cost-effective tools for assessing IR severity and their associations with NAFLD severity. However, evidence on their association with at-risk nonalcoholic steatohepatitis (NASH), a progressive phenotype characterized by hepatic fibrosis and inflammation, remains limited. This study aimed to evaluate the relationship between TyG related indices and at-risk NASH in a nationally representative NAFLD cohort.

**Methods:**

Using data from the 2017–2020 National Health and Nutrition Examination Survey (NHANES), adults with NAFLD diagnosed via vibration-controlled transient elastography (VCTE) were analyzed. At-risk NASH was defined using the FibroScan-AST (FAST) score. Five TyG related indices (TyG, TyG-WC, TyG-WHR, TyG-WtHR, TyG-BMI) were calculated and analyzed through weighted logistic regression, stratified analyses, restricted cubic spline (RCS), and receiver operating characteristic (ROC) analyses. Adjustments included demographics and clinical confounders.

**Results:**

Among participants, 10% exhibited at-risk NASH. Composite indices (TyG-WC, TyG-WHR, TyG-WtHR, TyG-BMI) each showed higher ORs than TyG alone in Model 3. TyG-WtHR exhibited the strongest association both as a continuous variable and across tertiles in Model 3, demonstrating a near-linear dose-response relationship. RCS analysis revealed no significant nonlinear relationships in composite indices, whereas the TyG index demonstrated a significant nonlinear association. Stratified analyses revealed elevated risks in males, middle-aged adults, and Non-Hispanic Black individuals. ROC analysis showed the discriminative performance of TyG-WC, while TyG-WtHR balanced sensitivity and specificity.

**Conclusion:**

This study demonstrated significant associations between TyG-related indices and at-risk NASH in NAFLD patients. These non-invasive, cost-effective biomarkers may facilitate for early risk stratification, guiding precision interventions, and optimizing clinical trial design to improve patient outcomes.

## 1 Introduction

Nonalcoholic fatty liver disease (NAFLD), affecting approximately 30% of the global population, is a rapidly growing chronic condition whose prevalence parallels the rise in obesity, type 2 diabetes mellitus (T2DM), and metabolic syndrome (MetS) (1, 2). It is characterized by excessive hepatic lipid accumulation in the absence of significant alcohol consumption, steatogenic medications, or viral infections and may progress from simple nonalcoholic fatty liver (NAFL) to nonalcoholic steatohepatitis (NASH), which can lead to fibrosis, cirrhosis, and hepatocellular carcinoma (HCC) (3). While viral hepatitis remains the leading cause of HCC, the growing incidence of NAFLD-related cirrhosis suggests a shift in its etiological factors (4, 5). As the more severe form of NAFLD, NASH has become the second most common indication for liver transplantation in 2019 and is the fastest increasing indication in the U.S (6). Currently, no FDA-approved pharmacotherapy exists for NAFLD/NASH, and the management focuses on addressing metabolic comorbidities and cardiovascular risk reduction (7). The presence of NASH, assessed histologically using the Brunt criteria and numerically via the NAFLD activity score (NAS) at trial entry, is often combined with fibrosis stage ≥2 (≥F2) to identify individuals at increased risk of severe liver-related complications who may benefit from clinical trials of new pharmacotherapies (8–10). Early identification of at-risk NASH patients with progressive fibro-inflammatory phenotypes and predicted therapeutic responsiveness is clinically imperative for preventing disease progression, improving patient outcomes and optimizing trial designs and treatment allocation (11, 12).

Cardio-metabolic risk factors, including insulin resistance (IR), obesity, dyslipidemia, and other related factors, are crucial in the development and progression of NAFLD and NASH (13, 14). IR, a key component of MetS, induces lipolysis in adipocytes, resulting in excessive free fatty acid production and ectopic lipid deposition, which is associated with NAFLD pathogenesis (15). The triglyceride-glucose (TyG) index, which combines fasting plasma glucose (FPG) and triglyceride (TG) levels, has emerged as a simple and cost-effective marker of IR (16–18). Elevated TyG index has been significantly linked to an increased risk of NAFLD, underscoring its potential as a useful and convenient diagnostic tool since it is easy to obtain (19, 20). Additionally, several modified TyG indices, such as TyG adjusted for waist circumference (TyG-WC), waist-to-hip ratio (TyG-WHR), waist-to-height ratio (TyG-WtHR), and body mass index (TyG-BMI), have been developed to more accurately assess IR severity and may be useful in identifying individuals at higher risk for NASH (21, 22).

Limited evidence exists regarding the relationship between IR indices and at-risk NASH. Hence, this study aimed to investigate the association between TyG related indices and at-risk NASH in U.S. adults with NAFLD. The findings may provide valuable insights into the early detection and prevention of NASH, aiding in the identification of high-risk populations for timely intervention or potential benefit in clinical trials of new therapies.

## 2 Materials and methods

### 2.1 Study population in NHANES

The data for this analysis were obtained from the publicly available National Health and Nutrition Examination Survey (NHANES) database. The NHANES study protocols were approved by the Research Ethics Review Board of the National Center for Health Statistics (NCHS), and informed consent was obtained from all participants. The study was exempt from institutional review board approval as it used de-identified, publicly available data.

There were totally 15,560 individuals participating in 2017–March 2020 (pre-pandemic) NHANES cycle. The study population was restricted to adults aged 18 years or older who successfully underwent complete liver elastography assessment using the FibroScan 502 Touch system (Echosens, Paris, France) with validated measurement outputs. Exclusion criteria included: (1) pregnancy status ascertained through both self-reporting from the Demographics Data and urine pregnancy test from the Laboratory Data, (2) pre-existing autoimmune hepatitis or liver cancer documented in the Medical Conditions component, (3) hepatitis B or C infection (defined by positive hepatitis B surface antigen or hepatitis C antibodies/RNA from the Laboratory Data), (4) significant alcohol consumption (an average of >30 g/day for men and >20 g/day for women) calculated from the Dietary Data (23, 24), (5) current or recent (within 30 days) use of steatogenic medications (e.g., amiodarone, methotrexate, and tamoxifen), with at least 6 months of continuous use prior to enrollment documented in the Prescription Medications component (25), and (6) incomplete essential data on relevant variables.

### 2.2 Definition and assessment of NAFLD and at-risk NASH in NHANES

Liver biopsy remains the gold standard for assessing hepatic steatosis and fibrosis staging in chronic liver diseases, including NAFLD, despite its invasive nature and high cost. From 2017 to March 2020, NHANES staff used the FibroScan 502 Touch device to perform vibration-controlled transient elastography (VCTE) on participants, which has the ability to assess hepatic steatosis through the controlled attenuation parameter (CAP) and fibrosis severity via liver stiffness measurement (LSM) (26, 27). A CAP cutoff value of 248 dB/m was used to identify hepatic steatosis in this study (28, 29). The FibroScan-AST (FAST) score, a validated non-invasive algorithm derived from CAP, LSM, and aspartate aminotransferase (AST) levels, demonstrates clinical utility for identifying at-risk NASH (FAST score ≥0.35) as established in prior studies (11, 26). FAST score was calculated using the following formula:


$$FAST\;score=\frac{e^{y}}{1+e^{y}}$$

$$y=-1.65+1.07\times \ln\left(LSM\right)+2.66\times 10^{-8}\times CAP^{3}-63.3\times AST^{-1}$$

**Figure 1:**
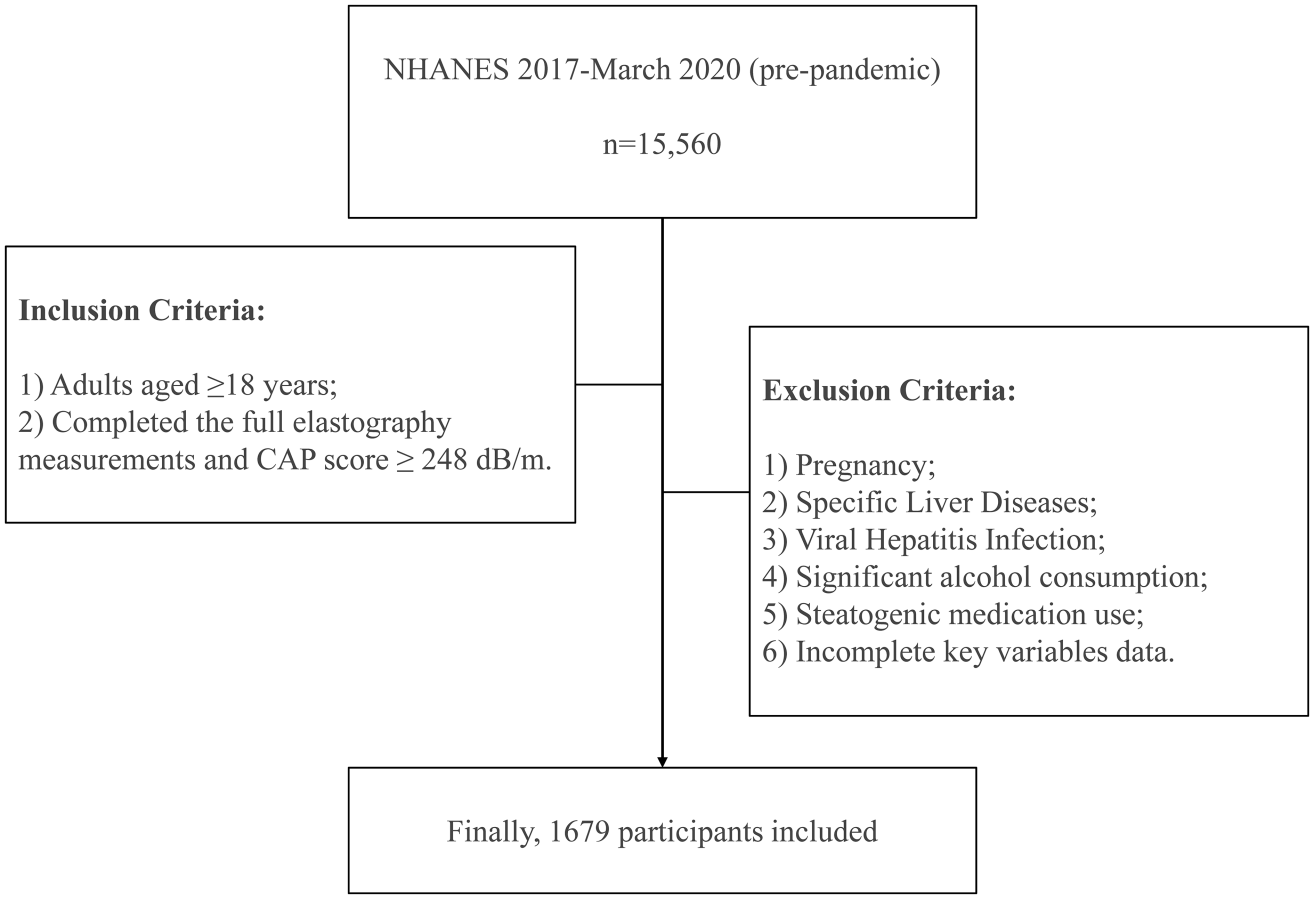
Flowchart of inclusion and exclusion criteria for NAFLD patients in the NHANES database.

Finally, this study consisted of 1679 participants. The detailed procedure for participant enrollment was described in **Figure 1**.

### 2.3 TyG related indices and covariates

Demographic data including gender, age, race, and education level were collected through standardized questionnaires from the Demographics Data. Anthropometric measurements from the Examination Data including body weight, height, waist circumference (WC), and hip circumference were obtained during standardized physical examinations at a mobile center; waist-to-hip ratio (WHR), body mass index (BMI, calculated as weight/height^2), and waist-to-height ratio (WtHR) were derived from these measurements. Laboratory parameters, including triglycerides (TG), fasting plasma glucose (FPG), platelet (PLT), alanine aminotransferase (ALT), AST, gamma-glutamyl transferase (GGT), and hemoglobin A1c (HbA1c), were measured using venous blood samples at baseline from the Laboratory Data.

TyG, TyG-WC, TyG-WHR, TyG-WtHR, and TyG-BMI indices were calculated using the following formulas:


WHR=WC(cm)/Hip Circumference(cm)

WtHR=WC(cm)/Height(cm)

TyG=ln[(TG(mg/dl)×FPG(mg/dl))/2]

TyG−WC=TyG×WC

TyG−WHR=TyG×WHR

TyG−WtHR=TyG×WtHR

TyG−BMI=TyG×BMI

Hypertension was defined as meeting any of the following criteria: 1) average systolic blood pressure (SBP) ≥140 mmHg or diastolic blood pressure (DBP) ≥90 mmHg based on three consecutive measurements at rest (30); 2) current use of antihypertensive medications; or 3) physician-diagnosed hypertension. T2DM diagnosis was established in participants without a history of Type 1 diabetes who fulfilled at least one of the following criteria: 1) physician-confirmed T2DM diagnosis; 2) active pharmacological treatment with glucose-lowering agents; 3) FPG ≥126 mg/dL (7.0 mmol/L); or 4) HbA1c ≥6.5% measured through standardized assays. Further details on covariate measurements are available in the NHANES documentation.

### 2.4 Statistical analysis

Analytical protocols for NHANES mandate the systematic incorporation of sampling weights and design variables in all statistical evaluations. This requirement stems from the complex sampling design employing multistage clustering and stratification with differential selection probabilities. Neglecting to properly adjust for these design elements may yield statistically invalid estimates. Fasting subsample weights were applied in accordance with NHANES analytic guidelines. For categorical variables, data were expressed as unweighted frequency counts and weighted percentages, and group differences were assessed using Rao-Scott adjusted chi-square tests. For continuous variables, data were summarized as median (interquartile range, IQR) and compared across groups via design-based Kruskal-Wallis rank-sum tests.

Weighted binary logistic regression models were constructed to evaluate associations between TyG related indices and at-risk NASH, with results expressed as odds ratio (OR) and 95% confidence interval (95%CI). Three models were employed: Model 1, unadjusted; Model 2, adjusted for demographic factors (age, gender, race, education level); Model 3, further adjusted for clinical variables (hypertension, T2DM, PLT, ALT, AST, GGT). TyG related indices were analyzed both as continuous variables and categorized into tertiles (T1-T3, T1 as reference). Moreover, weighted logistic regression models were used for stratified and interaction analyses based on gender, age, race, education level, hypertension, and T2DM status. Restricted cubic spline (RCS) analyses were performed to assess potential nonlinear associations. Predictive performance was assessed through receiver operating characteristic (ROC) curves, reporting area under the ROC curve (AUC). Statistical significance was defined as two-tailed *p* value<0.05. All statistical analyses were performed using the R software (R version 4.4.1).

## 3 Results

### 3.1 Participants’ baseline descriptions

This cross-sectional analysis enrolled 1,679 patients meeting the diagnostic criteria for NAFLD, stratified into 1,509 (90%, weighted) low-risk NASH patients and 170 (10%,weighted) at-risk NASH patients (**Table 1**). Compared to the low-risk group, the at-risk group demonstrated a significantly higher proportion of males and elevated prevalence of T2DM and hypertension. Significant intergroup differences were observed in laboratory markers, with PLT being lower ​and ALT, AST, and GGT levels being higher in the at-risk group (all *p* value< 0.001). All TyG related indices were significantly elevated in the at-risk group (all *p* value< 0.001). No significant differences were observed in age, race, or education level between groups (all *p* value> 0.05).

**Table 1:** Baseline features of the participants.

Characteristic	Overall	At-risk NASH		
	N = 1679 (100%)	No, N = 1509 (90%)	Yes, N = 170 (10%)	*P* value
Age (years)	53.0 (37.0, 64.0)	54.0 (37.0, 65.0)	47.0 (39.0, 60.0)	0.083
Age group				0.200
<40	419.0 (29.6%)	376.0 (29.8%)	43.0 (28.6%)	
40-60	593.0 (35.2%)	524.0 (34.2%)	69.0 (44.0%)	
>60	667.0 (35.2%)	609.0 (36.0%)	58.0 (27.4%)	
Gender				0.002
Female	835.0 (47.9%)	776.0 (49.7%)	59.0 (31.6%)	
Male	844.0 (52.1%)	733.0 (50.3%)	111.0 (68.4%)	
Race				0.300
Mexican American	264.0 (10.8%)	227.0 (10.5%)	37.0 (13.5%)	
Other race	440.0 (16.6%)	400.0 (16.7%)	40.0 (15.2%)	
Non-Hispanic White	618.0 (63.4%)	550.0 (63.2%)	68.0 (65.0%)	
Non-Hispanic Black	357.0 (9.3%)	332.0 (9.6%)	25.0 (6.3%)	
Education level				0.600
High school graduate or GED	408.0 (27.7%)	376.0 (28.2%)	32.0 (23.4%)	
Less than High school	342.0 (12.2%)	307.0 (12.0%)	35.0 (13.9%)	
Some college or above	929.0 (60.1%)	826.0 (59.8%)	103.0 (62.7%)	
Hypertension				0.047
No	782.0 (52.2%)	720.0 (53.5%)	62.0 (40.2%)	
Yes	897.0 (47.8%)	789.0 (46.5%)	108.0 (59.8%)	
T2DM				<0.001
No	1,165.0 (76.3%)	1,085.0 (78.8%)	80.0 (54.2%)	
Yes	514.0 (23.7%)	424.0 (21.2%)	90.0 (45.8%)	
PLT (1000 cells/uL)	237.0 (201.0, 278.0)	240.0 (204.0, 281.0)	209.0 (180.0, 256.0)	<0.001
ALT (U/L)	20.0 (14.0, 30.0)	19.0 (14.0, 27.0)	46.0 (31.0, 61.0)	<0.001
AST (U/L)	19.0 (16.0, 24.0)	19.0 (16.0, 23.0)	36.0 (27.0, 47.0)	<0.001
GGT (IU/L)	23.0 (16.0, 33.0)	21.0 (16.0, 30.0)	45.0 (29.0, 78.0)	<0.001
TyG	8.7 (8.3, 9.1)	8.7 (8.3, 9.1)	9.1 (8.7, 9.5)	<0.001
TyG-WC (cm)	933.8 (836.4, 1,057.8)	924.6 (831.5, 1,045.2)	1,100.9 (938.3, 1,237.0)	<0.001
TyG-WHR	8.4 (7.8, 9.0)	8.3 (7.7, 8.9)	9.3 (8.6, 9.8)	<0.001
TyG-WtHR	5.5 (5.0, 6.3)	5.5 (5.0, 6.2)	6.3 (5.6, 7.2)	<0.001
TyG-BMI (kg/m^2)	275.4 (240.4, 324.1)	271.3 (237.9, 317.8)	331.8 (281.8, 389.7)	<0.001

Data are displayed as the median (interquartile range) or unweighted frequency counts (weighted percentage) as appropriate. The design-based Kruskal-Wallis rank-sum test for continuous variables and the Rao-Scott adjusted chi-square test for categorical variables were used in this analysis.

GED, General Educational Development; T2DM, Type 2 Diabetes Mellitus; PLT, Platelet; ALT, Alanine Aminotransferase; AST, Aspartate Aminotransferase; GGT, Gamma-Glutamyl Transferase; TyG, Triglyceride-Glucose Index; TyG-WC, Triglyceride-Glucose Index to Waist Circumference; TyG-WHR, Triglyceride-Glucose Index to Waist-to-Hip ratio; TyG-WtHR, Triglyceride-Glucose Index to Waist-to-Height Ratio; TyG-BMI, Triglyceride-Glucose Index to Body Mass Index.

### 3.2 Associations between TyG related indices and at-risk NASH

Multivariable logistic regression analysis demonstrated differential associations between TyG related indices and at-risk NASH across three models (**Table 2**). As continuous variables, the TyG index showed statistically significant associations in Model 1 and Model 2, but lost statistical significance in Model 3. In contrast, all composite indices (TyG-WC, TyG-WHR, TyG-WtHR, TyG-BMI) maintained statistically significant associations across three models. Notably, TyG-WtHR showed the strongest association as a continuous variable in Model 3 (OR=3.730, 95% CI:1.957-7.110, ​*p* value<0.01). When stratified by tertiles, TyG-WC demonstrated a striking 18.517-fold increased odds in the T3 versus T1. TyG-WtHR retained statistically significant graded associations in Model 3 (*p* for trend<0.05).

**Table 2:** Associations between TyG related indices and at-risk NASH in the multiple logistic regression model.

Parameters	Model1 OR (95% CI)	*p* value	Model2 OR (95% CI)	*p* value	Model3 OR (95% CI)	*p* value
TyG	2.343 (1.617,3.396)	***	1.764 (1.212, 2.568)	**	1.304 (0.781, 2.178)	*ns*
Tertile						
T1	Reference		Reference		Reference	
T2	2.261 (1.047,4.884)	*	1.975 (0.798, 4.892)	*ns*	2.138 (0.494, 9.259)	*ns*
T3	4.524 (2.189,9.349)	***	3.153 (1.381, 7.198)	*	2.613 (0.694, 9.842)	*ns*
*p* for trend		***		*		*ns*
TyG-WC	1.006 (1.004,1.007)	***	1.006 (1.003, 1.008)	***	1.008 (1.004, 1.012)	**
Tertile						
T1	Reference		Reference		Reference	
T2	3.892 (1.439, 10.529)	**	3.694 (1.280, 10.664)	*	6.858 (0.865, 54.394)	*ns*
T3	9.900 (4.526, 21.654)	***	8.213 (3.086, 21.859)	***	18.517 (2.088, 164.195)	*
*p* for trend		***		***		*
TyG-WHR	2.383 (1.859, 3.055)	***	2.117 (1.579, 2.837)	***	1.977 (1.150, 3.397)	*
Tertile						
T1	Reference		Reference		Reference	
T2	1.929 (0.821, 4.531)	*ns*	1.670 (0.674, 4.142)	*ns*	1.281 (0.249, 6.590)	*ns*
T3	7.399 (3.161, 17.318)	***	5.165 (1.896, 14.071)	**	4.094 (0.747, 22.445)	*ns*
*p* for trend		***		**		*ns*
TyG-WtHR	2.180 (1.765, 2.692)	***	2.441 (1.718, 3.467)	***	3.730 (1.957, 7.110)	**
Tertile						
T1	Reference		Reference		Reference	
T2	3.118 (1.190, 8.169)	*	3.328 (1.159, 9.556)	*	5.886 (1.049, 33.035)	*
T3	7.302 (3.636, 14.666)	***	7.578 (2.893, 19.849)	***	15.435 (2.106, 113.144)	*
*p* for trend		***		***		*
TyG-BMI	1.010 (1.007, 1.013)	***	1.011 (1.007, 1.016)	***	1.016 (1.009, 1.023)	***
Tertile						
T1	Reference		Reference		Reference	
T2	2.081 (0.817, 5.300)	*ns*	2.126 (0.783, 5.771)	*ns*	2.718 (0.634, 11.643)	*ns*
T3	4.697 (1.941, 11.367)	**	4.719 (1.619, 13.754)	**	9.407 (1.991, 44.455)	**
*p* for trend		**		**		**

Model 1: unadjusted; Model 2: adjusted for demographic factors (age, gender, race, education level); Model 3: further adjusted for clinical variables (hypertension, T2DM, PLT, ALT, AST, GGT). *p* for trend was calculated by modeling the tertile rank as a continuous variable.

OR, odds ratio; 95% CI, 95% confidence intervals; TyG, Triglyceride-Glucose Index; TyG-WC, Triglyceride-Glucose Index to Waist Circumference; TyG-WHR, Triglyceride-Glucose Index to Waist-to-Hip ratio; TyG-WtHR, Triglyceride-Glucose Index to Waist-to-Height Ratio; TyG-BMI, Triglyceride-Glucose Index to Body Mass Index.

* *p* value< 0.05; ** *p* value< 0.01; *** *p* value< 0.001; *ns*, *p* value> 0.05.

### 3.3 Stratified analysis

**Figure 2:**
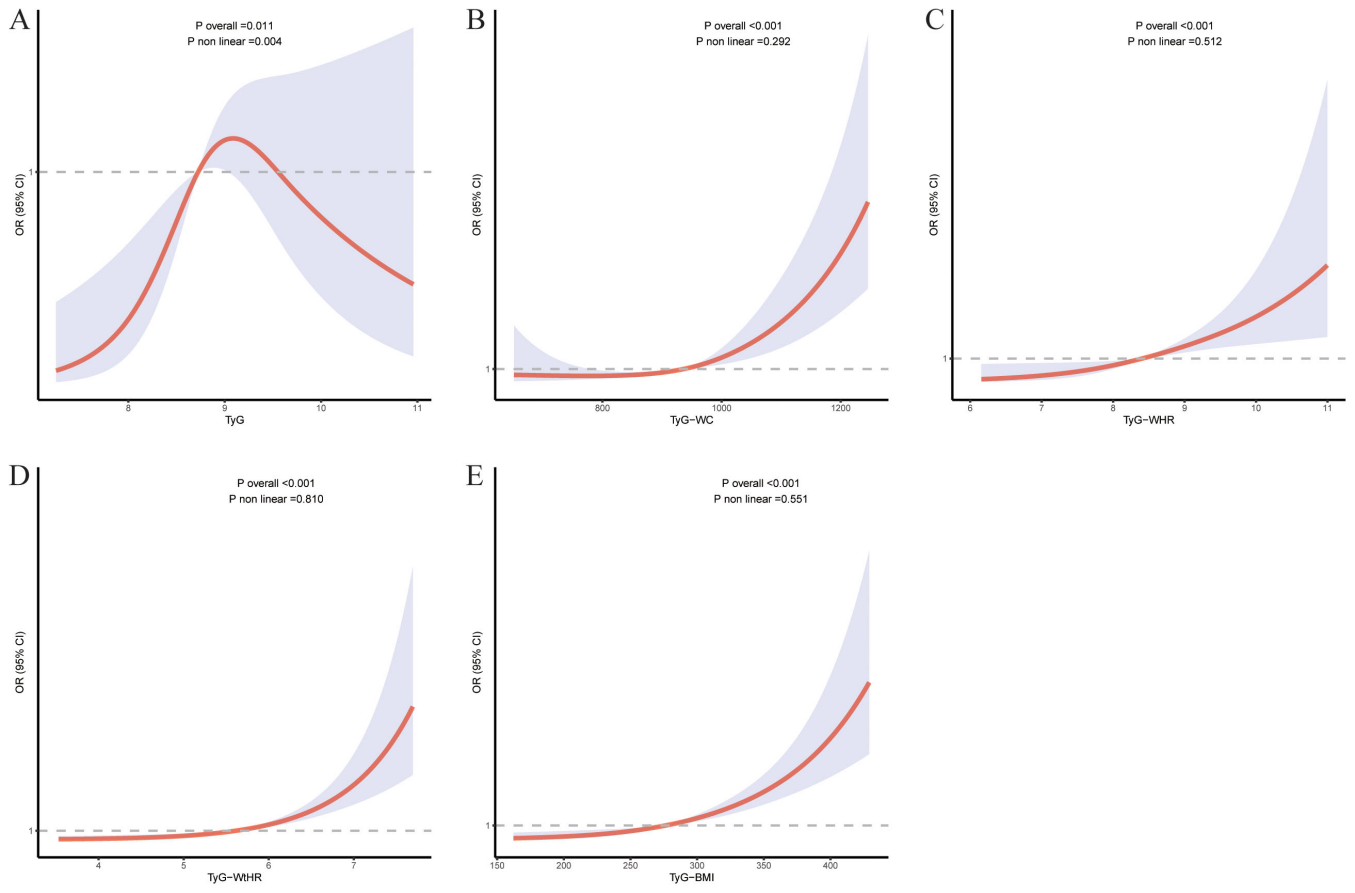
The RCS for the relationship between TyG related indices and at-risk NASH. The RCS curves for the relationship between TyG index **(A)**, TyG-WC **(B)**, TyG-WHR **(C)**, TyG-WtHR **(D)**, TyG-BMI **(E)** and at-risk NASH. The solid lines represent the multivariate-adjusted ORs, while the dashed lines depict the 95% CIs obtained from RCS regression.

Stratified analyses demonstrated significant effect modification in the associations between TyG related indices and at-risk NASH across demographic and clinical subgroups (**Figure 2**). The TyG index exhibited stronger associations in non-hypertensive and non-T2DM compared to their counterparts, with significant interaction effects (*p* for interaction=0.021 and 0.008, respectively). Gender disparities were evident for TyG-WtHR, with males showing markedly higher effect than females, though the interaction did not reach statistical significance (*p* for interaction=0.115). Age stratification revealed amplified effects in middle-aged populations, particularly for TyG-WHR, TyG-WC, and TyG-WtHR. Racial disparities emerged as clinically significant modifiers. Non-Hispanic Black individuals exhibited heightened risks for TyG-WHR and TyG-WtHR, while the Other race subgroup showed stronger associations for TyG and TyG-WtHR. Educational attainment significantly modified TyG-WC associations (*p* for interaction=0.020), with high school graduates/General Educational Development (GED) holders showing the strongest effect.

### 3.4 RCS analysis

**Figure 3:**
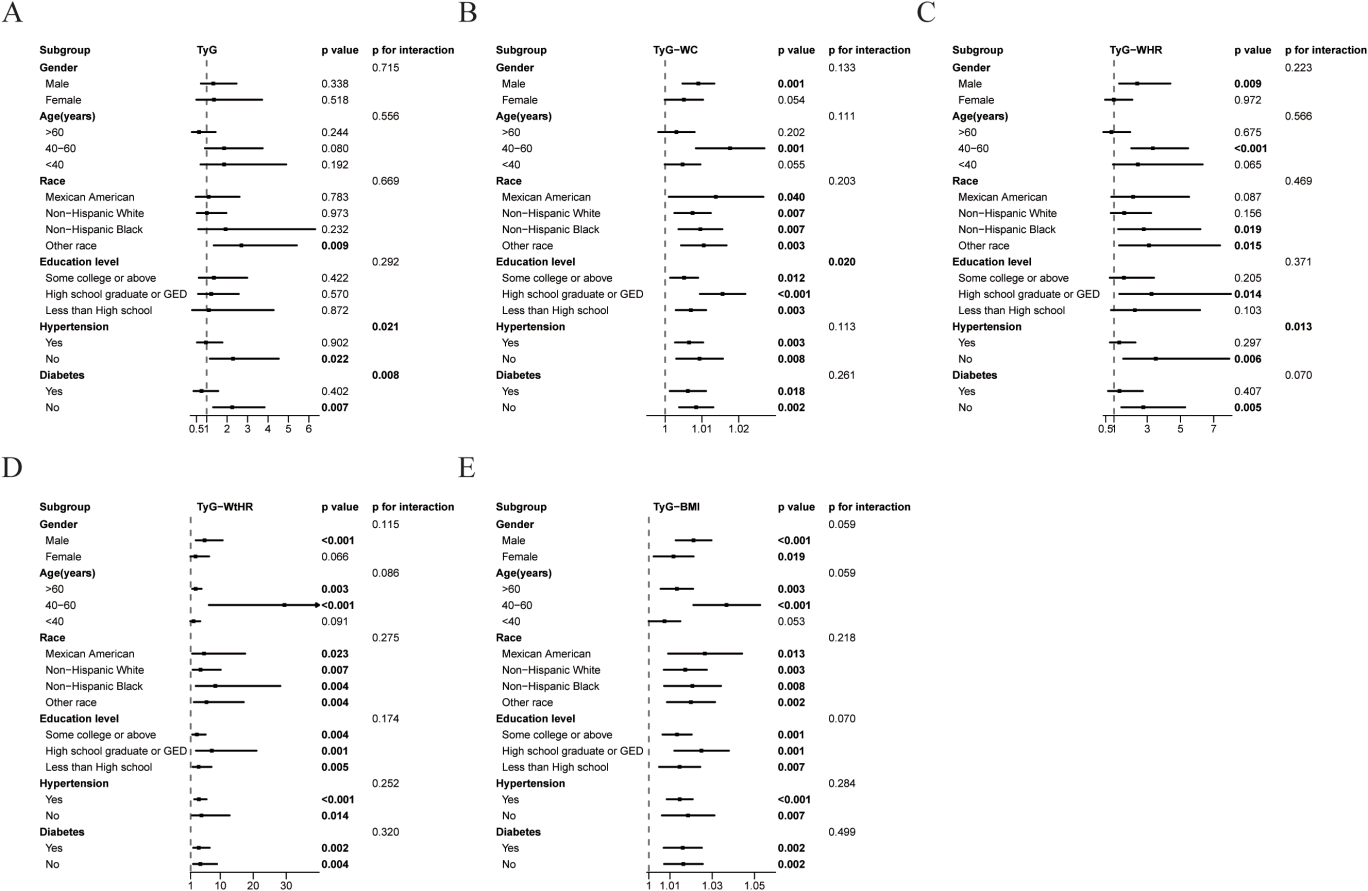
Association between TyG related indices and at-risk NASH in different subgroups. Subgroups analysis of the relationship between TyG index **(A)**, TyG-WC **(B)**, TyG-WHR **(C)**, TyG-WtHR **(D)**, TyG-BMI **(E)** and at-risk NASH. Except for the stratification component itself, each stratification factor was adjusted for gender, age, race, education level, ALT, AST, GGT, hypertension, and T2DM status.

RCS analyses revealed distinct association patterns across TyG related indices. All composite indices demonstrated statistically significant overall associations with at-risk NASH, while showing nonsignificant nonlinearity (*p*-nonlinearity range: 0.292-0.810). In contrast, TyG exhibited both significant overall (*p*-overall=0.011) and nonlinear associations (*p*-nonlinearity=0.004), suggesting a curvilinear relationship with at-risk NASH (**Figure 3**).

### 3.5 ROC analysis

**Figure 4:**
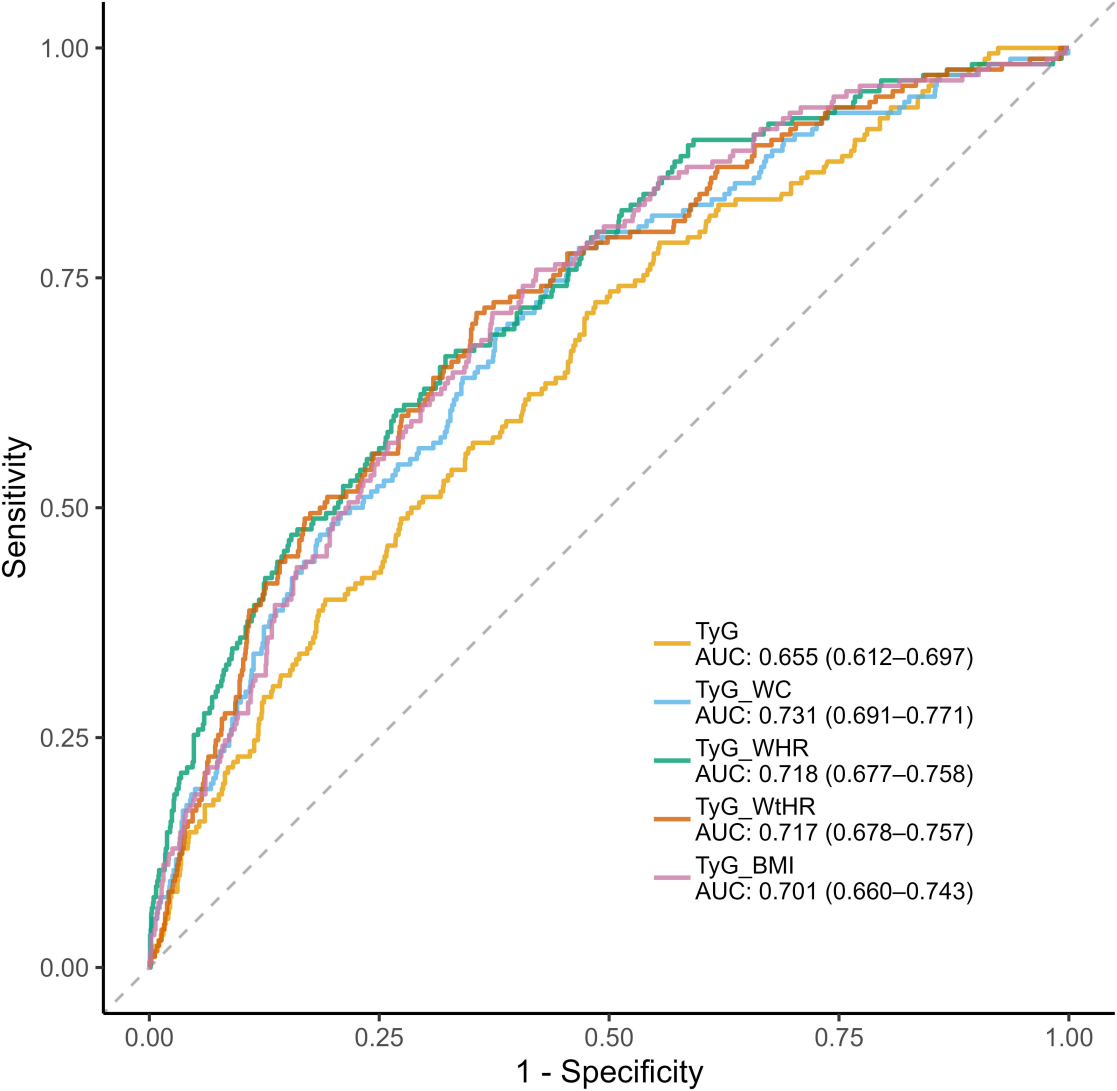
ROC curve analysis of TyG related indices for diagnosing at-risk NASH.

The diagnostic performance of TyG related indices was systematically evaluated through ROC analysis (**Figure 4**). Composite indices integrating anthropometric measures demonstrated superior discriminative capacity compared to the basic TyG index, with TyG-WC achieving the highest AUC (0.731, 95%CI:0.691-0.771), followed by TyG-WHR (AUC=0.718, 95%CI:0.677-0.758) and TyG-WtHR (AUC=0.717, 95%CI:0.678-0.757). TyG-WHR demonstrated the optimal balance between sensitivity and specificity (Youden Index=0.356), with 71.2% sensitivity and 64.4% specificity at its optimal cutoff. In contrast, TyG index showed high sensitivity (72.4%) but poor specificity (51.5%). The composite indices generally showed superior accuracy (64.4-67.8%) compared to TyG index (53.9%), with TyG-WC achieving the highest overall accuracy (67.8%) (**Table 3**).

**Table 3:** ROC analysis of diagnostic performance of TyG related indices for at-risk NASH.

Index	AUC (95% CI)	Youden Index	Sensitivity	Specificity	Accuracy
TyG	0.655 (0.612–0.697)	0.238	0.724	0.515	0.539
TyG-WC	0.731 (0.691–0.771)	0.343	0.665	0.678	0.678
TyG-WHR	0.718 (0.677–0.758)	0.356	0.712	0.644	0.674
TyG-WtHR	0.717 (0.678–0.757)	0.339	0.712	0.627	0.644
TyG-BMI	0.701 (0.660–0.743)	0.319	0.776	0.542	0.567

AUC, area under the receiver operating characteristic curve; 95% CI, 95% confidence intervals; TyG, Triglyceride-Glucose Index; TyG-WC, Triglyceride-Glucose Index to Waist Circumference; TyG-WHR, Triglyceride-Glucose Index to Waist-to-Hip ratio; TyG-WtHR, Triglyceride-Glucose Index to Waist-to-Height Ratio; TyG-BMI, Triglyceride-Glucose Index to Body Mass Index.

## 4 Discussion

The TyG index has been established as a noninvasive predictor for occurrence and severity of NAFLD in previous studies (31, 32). This study extends existing evidence by demonstrating the differential utility of TyG-related indices in identifying at-risk NASH within a nationally representative NAFLD cohort. The findings of this study hold substantial clinical relevance for early identification and management of at-risk NASH. While the TyG index lost statistical significance after full adjustment, the composite indices were significantly associated with at-risk NASH in NAFLD patients, underscoring the critical interplay between IR and visceral adiposity associated with at-risk NASH progression. Notably, TyG-WtHR maintained a strong association even after adjusting for a range of demographic and clinical covariates, suggesting its potential utility as a predictor of at-risk NASH in NAFLD patients. The strong association between TyG-WtHR and at-risk NASH highlights its utility as a non-invasive stratification tool in routine clinical practice. As a cost-effective alternative to liver biopsy, TyG-WtHR requires only routine measurements of TG, FPG, WC, and height, offering superior accessibility for population-level screening. RCS analysis of TyG-WtHR revealed a monotonic, near-linear increase in the likelihood of at-risk NASH, aligning with tertile analyses. TyG-WtHR maintained statistical significance across all models, indicating its dual capacity to reflect both IR and visceral adiposity, and showing superior specificity to BMI for central fat distribution. These findings collectively highlight the synergistic role of metabolic dysfunction and obesity in NASH pathophysiology, emphasizing the clinical utility of TyG-WtHR for early risk identification and stratification. Visceral adipose tissue was associated with increased hepatic lipotoxicity via pro-inflammatory adipokine secretion and lipogenesis linked to hyperinsulinemia (33). These results are consistent with recent research indicating that composite indices integrating metabolic and anthropometric parameters more accurately capture the multifactorial progression of NASH/NAFLD (34, 35).

Beyond the near-linear relationship observed for the TyG-WtHR, the RCS analysis of the TyG index revealed a statistically significant inverted U-shaped association with at-risk NASH. Specifically, the risk rose progressively across moderate TyG levels, reached a plateau at higher values, and then declined. This pattern may reflect the effects of more intensive metabolic treatments in patients with higher TyG, or survival bias among those with extreme dysmetabolism. These findings underscore the necessity of considering nonlinear modeling when establishing clinically relevant TyG thresholds.

​Stratified analyses revealed clinically effect modifications, advocating for personalized risk stratification. In hypertensive and diabetic subgroups, the association between TyG and at-risk NASH was attenuated, implying that more complex metabolic disturbances may be more closely associated with liver injury than IR alone (15). Notably, racial disparities demonstrated significant effect modification (36, 37), with Non-Hispanic Black individuals showing a pronounced 8.5-fold elevated risk associated with TyG-WtHR. Younger, non-diabetic males may derive particular benefit from TyG related indices screening. Middle-aged populations exhibited the most pronounced risk amplification, particularly for TyG-WtHR, suggesting age-specific thresholds for clinical intervention. This disparity may reflect gender and age specific differences in visceral fat distribution or estrogen-mediated hepatoprotection (37–40). The persistent statistical significance of TyG-WtHR across most subgroups underscores its robustness as a risk stratification tool. Clinicians could prioritize patients with elevated TyG-WtHR for advanced diagnostics or lifestyle interventions. These findings underscore the necessity of population-stratified risk assessment in NAFLD patients management.

The diagnostic performance evaluation revealed critical insights into clinical applications of TyG related indices. Composite indices combining anthropometric measures demonstrated significantly higher discriminative performance than the basic TyG index, consistent with their maintained statistical significance in the fully adjusted logistic regression models. Clinically, balanced sensitivity and specificity of TyG-WC supports screening applications, while the monotonic dose-response relationship of TyG-WtHR favors dynamic risk stratification and treatment response monitoring.

Several strengths of this study are noted. First, this study used a nationally representative database, coupled with the application of complex sampling weights recommended by NHANES protocols, to make the results reliable. Second, hepatic steatosis and at-risk NASH were measured by VCTE, which is more sensitive and accurate than ultrasound (41). Third, various analytical methods, including logistic regression, RCS, and ROC, were systematically employed to evaluate the association between TyG related indices and at-risk NASH, clarifying the predictive value of these indices.

However, it is important to note some limitations. First, many diagnoses in the analysis including NAFLD and at-risk NASH are based on NHANES database algorithms rather than confirmed clinical or histological reports, which may lead to diagnostic misclassification and information bias. Second, residual confounding by unmeasured factors cannot be excluded. Third, the cross-sectional nature of the NHANES data limits causal inference. Although strong associations were observed, it remains unclear whether elevated TyG related indices predict NASH progression or simply reflect concurrent metabolic and inflammatory status. Therefore, prospective cohort studies or randomized interventions are needed to validate these relationships over time and clarify causality.

Additionally, future research should also compare TyG related indices directly to liver histology to confirm their diagnostic accuracy, evaluate whether longitudinal changes in TyG-WtHR correlate with histological improvement, and investigate the added value of integrating TyG related indices with emerging biomarkers. Further mechanistic studies exploring how IR and visceral adiposity are associated with hepatocyte apoptosis and fibrogenesis are also warranted.

## 5 Conclusion

In conclusion, this cross-sectional analysis revealed multidimensional associations between TyG related indices and at-risk NASH in NAFLD patients. These non-invasive, cost-effective indices may facilitate early risk stratification in metabolically complex populations and support precision intervention strategies, with potential to inform and optimize clinical trial design and ultimately improve patient outcomes.

## Data Availability

The raw data supporting the conclusions of this article will be made available by the authors, without undue reservation.
